# The efficacy and safety of anti-PD-1/PD-L1 in treatment of glioma: a single-arm meta-analysis

**DOI:** 10.3389/fimmu.2023.1168244

**Published:** 2023-04-14

**Authors:** Yi-Fan Zeng, Xin-Yu Wei, Qi-Hao Guo, Si-Yu Chen, Sheng Deng, Zheng-Zheng Liu, Zhi-Cheng Gong, Wen-Jing Zeng

**Affiliations:** ^1^Department of Cardiovascular Surgery, The Second Xiangya Hospital, Central South University, Changsha, Hunan, China; ^2^Department of Pharmacy, Xiangya Hospital, Central South University, Changsha, Hunan, China; ^3^National Clinical Research Center for Geriatric Disorders, Xiangya Hospital, Central South University, Changsha, Hunan, China; ^4^Department of Pharmacy, Shengjing Hospital of China Medical University, Shenyang, China; ^5^Department of Oncology, Xiangya Hospital, Central South University, Changsha, Hunan, China

**Keywords:** glioma, PD- 1/L1, drug safety, drug effect evaluation, meta - analysis

## Abstract

**Objective:**

This meta-analysis aimed to evaluate the efficacy and safety of PD-1/PD-L1 inhibitors in patients with glioma.

**Methods:**

PubMed, EMBASE, Web of Science, and the Cochrane library were searched from inception to January 2023 without language restriction. Primary outcomes included overall survival (OS), progression-free survival (PFS), objective response rate (ORR), and adverse events (AEs). The risk of bias was assessed by subgroup analysis, sensitivity analysis, and publication bias, including funnel plot, Egger’s test, and Begg’s test.

**Results:**

A total of 20 studies involving 2,321 patients were included in this meta-analysis. In the analysis of the included phase III clinical trials, the forest plot showed that PD-1/PD-L1 inhibitors did not improve the OS (HR=1.15, 95% CI: 1.03-1.29, P=0.02, I^2 ^= 14%) and PFS (HR=1.43, 95% CI: 1.03-1.99, P=0.03, I^2 ^= 87%). In the single-arm analysis, the forest plot demonstrated that the 6-month OS was 71% (95% CI: 57%-83%, I^2 ^= 92%), 1-year OS was 43% (95% CI: 33%-54%, I^2^ = 93%), and the 2-year OS was 27% (95% CI: 13%-44%, I^2 ^= 97%). The pooled estimate of the median OS was 8.85 months (95% CI: 7.33-10.36, I^2 ^= 91%). Furthermore, the result indicated that the 6-month PFS was 28% (95% CI: 18%-40%, I^2 ^= 95%), 1-year PFS was 15% (95% CI: 8%-23%, I^2^ = 92%), and the 18-month PFS was 10% (95% CI: 3%-20%, I^2 ^= 93%). The pooled estimate of the median PFS was 3.72 months (95% CI: 2.44-5.00, I^2 ^= 99%). For ORR, the pooled estimate of ORR was 10% (95% CI: 2%-20%, I^2^ = 88%). We further analyzed the incidence of PD-1/PD-L1 inhibitor-related AEs, and the pooled incidence of AEs was 70% (95% CI: 58%-81%, I^2 ^= 94%). The incidence of AEs ≥ grade 3 was 19% (95% CI: 11%-30%, I^2 ^= 94%). The funnel plot for the median PFS and median OS was symmetric with no significant differences in Egger’s test and Begg’s test. The sensitivity analysis revealed that our results were stable and reliable.

**Conclusion:**

The results of this meta-analysis suggest that anti-PD-1/PD-L1 therapy is relatively safe but could not prolong survival in glioma. More randomized controlled trials are needed to confirm our results.

**Systematic review registration:**

https://www.crd.york.ac.uk/prospero/, identifier CRD42023396057.

## 1 Introduction

Glioma is the most common primary malignant brain tumor, accounting for approximately 27% of central nervous system tumors (1). The CBTRUS statistical report shows that the incidence of glioblastoma (GBM) is age-related, with 0.15/100,000 in children aged 0-14 years, 0.48/100,000 in people aged 15-39 years, and 6.96/100,000 in adults over 40 years old (2). Gliomas are classified by World Health Organization (WHO) as grades 1-4 according to the degree of malignancy, among which GBM is the most malignant, with a median survival of about 15 months (3). The current standard treatment for glioma is to maximally remove the tumor by surgical resection, combined with radiotherapy (RT) and chemotherapy with temozolomide (TMZ), under the premise of ensuring complete function (4). However, due to the infiltrative growth of malignant glioma, it is difficult to completely remove the tumor and easy to occur with radiotherapy and chemotherapy resistance, resulting in high recurrence and mortality with poor survival prognosis in patients with glioma (5). Therefore, novel therapeutic strategies are urgently needed to reduce the recurrence rate and improve the prognosis of glioma.

Programmed death ligand 1 (PD-L1) is an immune checkpoint that binds to the receptor PD-1 expressed on immune cells, mainly T cells, to modulate the immune response (6). Anti-PD-1/PD-L1 is currently the primary treatment for certain cancers because it is upregulated in various tumors, including melanoma, non-small cell lung cancer, and colorectal cancer, and is involved in tumor immune escape (7–12). Multiple studies have shown that PD-L1 is upregulated in glioma, and PD-1/PD-L1 inhibitors in combination with other therapies significantly prolong the survival time in mice, showing positive therapeutic potential (12–15). However, in clinical trials, whether PD-1/PD-L1 inhibitors improve the prognosis of patients is still controversial. Currently commonly used PD-1/PD-L1 inhibitors in clinical trials for glioma include nivolumab, atezolizumab, camrelizumab, pembrolizumab, and durvalumab (16, 17). This meta-analysis was conducted to evaluate the efficacy and safety of PD-1/PD-L1 inhibitors in patients with glioma.

## 2 Methods

### 2.1 Search strategy

We retrieved relevant literature from four databases including Embase, PubMed, Web of Science, and the Cochrane library from their inception to January 2023 without language restriction. The keywords used were “retifanlimab”, “balstilimab”, “immune checkpoint inhibitor”, “nivolumab”, “pembrolizumab”, “cemiplimab”, “sintilimab”, “camrelizumab”, “atezolizumab”, “avelumab”, “durvalumab” and “glioma”.

### 2.2 Study selection

This meta-analysis has been registered in PROSPERO (ID: CRD42023396057). All the following steps including study selection, data extraction, and quality assessment were conducted in Covidence.

Inclusion criteria (1): Study type: randomized controlled trial (RCT), prospective clinical trials, retrospective cohort studies, prospective case-control studies, and case series (n>10); (2) Diagnose: patients diagnosed with primary or recurrent glioma; (3) Drug: anti-PD1/PD-L1 therapy regardless of dosage, administration method, and duration; and (4) Data: detail data of primary outcomes. Primary outcomes include overall survival (OS), progression-free survival (PFS), objective response rate (ORR), and adverse events (AEs).

Exclusion criteria: (1) *in vitro* or animal experiments; (2) unable to extract the exact data in the article; and (3) conference abstract.

### 2.3 Data extraction

Two authors independently filtrated the titles and abstracts of relevant articles based on the established inclusion and exclusion criteria and comprehensively reviewed the literature with high relevance, and then extracted data from the articles that finally met the research criteria. This process was then verified by a third researcher, and any disagreements therein were adjudicated through group discussion.

Information extracted from the included studies as follows: (1) Study information: the first author, publication year, country, and study design; (2) Patient information: number of participants, gender, and age; (3) Treatment information: drug of PD-1/PD-L1 inhibitor, route of administration, dose, duration, and combination of treatment; (4) Primary outcomes: OS, PFS, ORR, and AEs.

### 2.4 Quality assessment

The quality assessment of included RCT was independently evaluated by two authors using the Cochrane Risk of Bias Tool (ROB2). Retrospective cohort studies, prospective case-control studies, and case series (n>10) were assessed by the Newcastle-Ottawa Scale (NOS).

### 2.5 Statistical analysis

The Cochran Q test and the I^2^ statistics were used to evaluate the heterogeneity. Different effect models were selected based on I^2^ statistics. When the I^2^ value is over 50%, the random effect model was used. On the contrary, the fixed effect model was used. Subgroup analyses were conducted to probe the source of the heterogeneity. Publication bias analysis was performed by funnel plot, Egger’s test, and Begg’s test. All the analyses above were conducted by R (Version 4.2.2). *P*<0.05 indicated a statistically significant difference.

## 3 Results

### 3.1 Studies selection and characteristics

**Figure 1:**
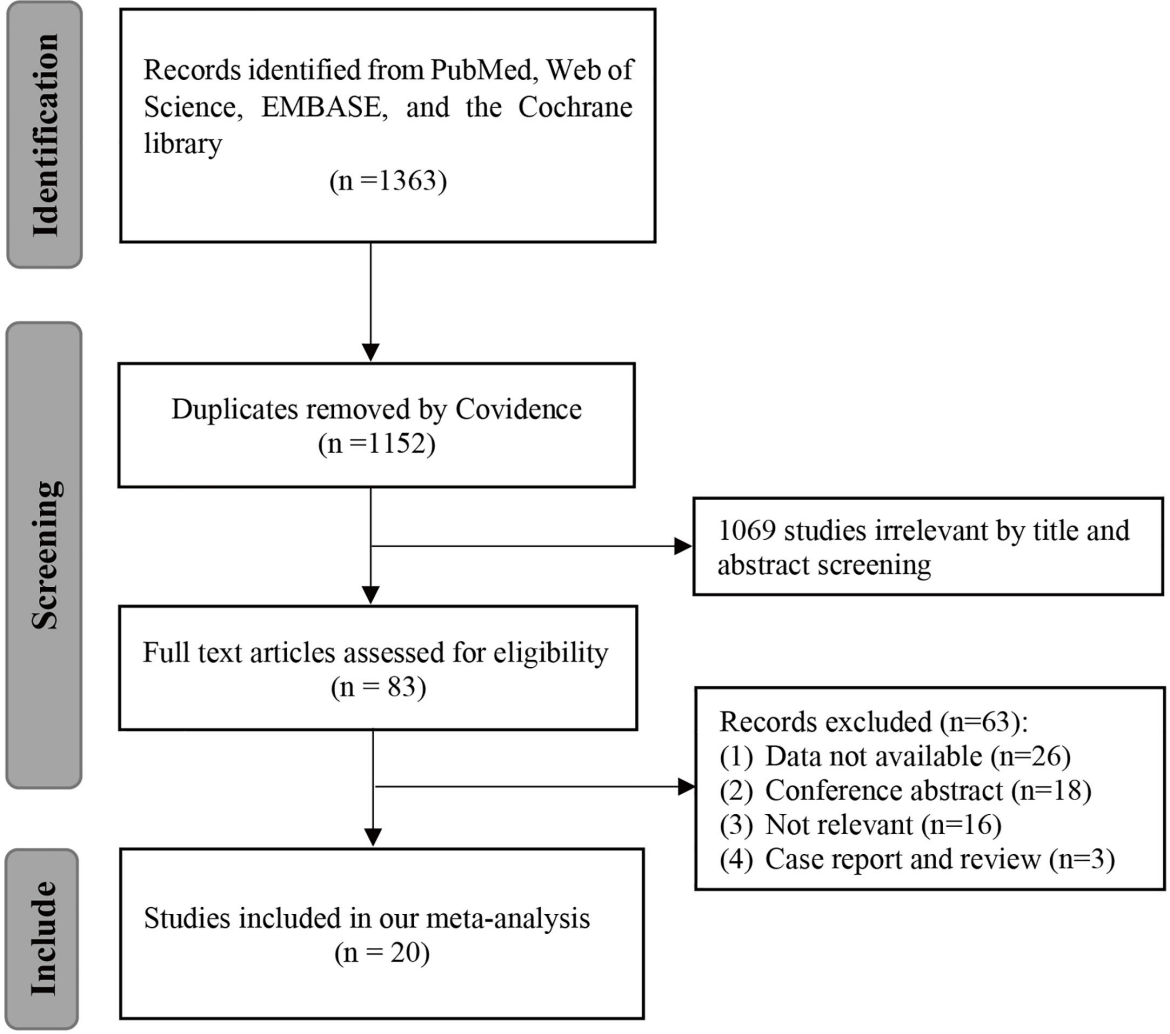
Flow diagram of study selection.

After a preliminary search, we found 1,363 articles in four databases, including 1,037 articles from Embase, 93 articles from PubMed, 201 articles from Web of Science, and 32 articles from the Cochrane library. After excluding 211 duplicates using Covidence, two independent researchers screened the titles and abstracts of the remaining 1,152 articles and excluded 1,069 unrelated studies. Sixty-three articles were then excluded after reading the full text of the remaining 83 articles, leaving a total of 20 articles with 2,321 patients being included in our meta-analysis (18–37). The PRISMA flow chart was shown in **Figure 1**.

Concerning PD-1/PD-L1 inhibitors, 10 studies utilized nivolumab, 8 studies utilized pembrolizumab, and 1 each utilized avelumab, atezolizumab, and durvalumab. As regards study design, there were 4 phase I studies, 6 phase II studies, 3 phase III studies, and 7 retrospective studies. The publication year ranged from 2015 to 2022. The detailed baseline characteristics of included clinical trials in this meta-analysis were shown in **Table 1**.

**Table 1:** Baseline characteristics of included studies.

Author	Year	Design (register number)	No. patients	Male (%)	Age (years)	Diagnose	PD-1/PD-L1 inhibitors	Dose
Aoki et al.	2021	Phase II (JapicCTI152967)	50	34 (68)	< 65 (n=37), 65-<75 (n=12), ≥ 75 (n=1)	recurrent GBM and gliosarcoma	Nivo	3mg/kg, i.v., Q2w
Awada et al.	2020	Phase II (NCT03291314)	54	34 (62.9)	55(19-75)	recurrent GBM	Avelumab	10mg/kg, i.v., Q2w
Blumenthal et al.	2015	Retrospective study	22	8 (36.4)	Adults: 38.5 (25–71) Children: 5 (3–7)	recurrent advanced primary CNS tumors	Pembrolizumab	Adults:150mg, i.v., Q3w; Children: 50mg, i.vi, Q3w
Chamberlain et al.	2017	Retrospective case series	16	11 (68.8)	59.5 (52-72)	recurrent GBM	Nivo	3mg/kg, i.v., Q2w
CheckMate143	2020	Phase III RCT (NCT02017717)	Nivo:184 BEV:185	235 (63.7)	Nivo: 55.5 (22-77) BEV: 55.0 (22-76)	recurrent GBM or gliosarcoma	Nivo	3mg/kg, i.v., Q2w
CheckMate498	2023	Phase III RCT (NCT02617589)	Nivo+RT:280 TMZ+RT:280	365 (65.2)	Nivo+RT: 59.5 (18-83) TMZ+RT: 56.0 (23-81)	GBM or gliosarcoma	Nivo	240mg, i.v., Q2w (8 doses) +480mg, i.v., Q4w
CheckMate548	2022	Phase III RCT (NCT02667587)	Nivo+RT+TMZ: 358 Placebo+RT+TMZ: 358	402 (56.1)	Nivo+RT+TMZ: 60.0 (24-79) Placebo+RT+TMZ: 60.0 (18-81)	GBM	Nivo	240mg, i.v., Q2w (8 doses) +480mg, i.v., Q4w
deGroot et al.	2020	Phase II (NCT02337686)	15	NA	NA	recurrent GBM	Pembrolizumab	200mg, i.v., Q3w
Duerinck et al.	2021	Phase 1 (NCT03233152)	27	17 (63.0)	55 (38–74)	GBM	Nivo	10mg/kg, intracerebral when surgery, then 10mg/kg, i.v., Q2w
KeyNote028	2020	Phase I (NCT02054806)	26	14 (53.8)	55.5 (33-76)	recurrent and no/failed prior standard therapy GBM	Pembrolizumab	10mg/kg, i.v., Q2w
Kline et al.	2018	Retrospective study	31	10(32.3)	NA	recurrent diffuse intrinsic pontine glioma	Nivo	3mg/kg, i.v., Q2w
Kurz et al.	2018	Retrospective study	31	12 (38.7)	49.8(19-82)	recurrent HGG	Pembrolizumab or Nivo	Pembrolizumab: 2 mg/kg, i.v., Q3w; Nivolumab: 3 mg/kg, i.v., Q2w
Lombardi et al.	2020	Observational pilot study	13	7 (53.8)	43 (21-65)	recurrent HGG	Pembrolizumab	200mg, i.v., Qw
Lukas et al.	2018	Phase Ia (NCT01375842)	16	13 (81.3)	52 (31-75)	recurrent GBM	Atezolizumab	1200mg, i.v., Q3w
Mantica et al.	2018	Retrospective study	50	30 (60)	55 (25-75)	recurrent HGG	Nivo	3mg/kg, i.v., Q2w
Nayak et al.	2022	Phase II (NCT02336165)	159	106 (66.7)	56.0 (22-77)	recurrent GBM	Durvalumab	10mg/kg, i.v., Q2w
Nayak et al.	2021	Phase II (NCT02337491)	80	54 (67.5)	53 (42–60)	recurrent GBM	Pembrolizumab	200mg, i.v., Q3w
Reiss et al.	2017	Retrospective study	25	11 (44)	49(30-72)	recurrent HGG	Pembrolizumab	3 doses (range 1–14) (NA)
Sahebjam et al.	2020	Phase I (NCT02313272)	32	21 (65.6)	BEV Naive: 55.5 (22–68) BEV Resistant: 49.5 (27–61)	recurrent GBM or anaplastic astrocytoma	Pembrolizumab	100mg- 200mg, i.v., Q3w
Schalper et al.	2019	Phase II (NCT02550249)	29	19 (65.5)	54 (33-73)	recurrent GBM	Nivo	3mg/kg, i.v., Q2w

Nivo, nivolumab; BEV, bevacizumab; TMZ, temozolomide; RT, radiotherapy; CNS, central nervous system; GBM, glioblastoma; HGG, high-grade glioma.

### 3.2 Overall survival and progression‐free survival in RCT

**Figure 2:**
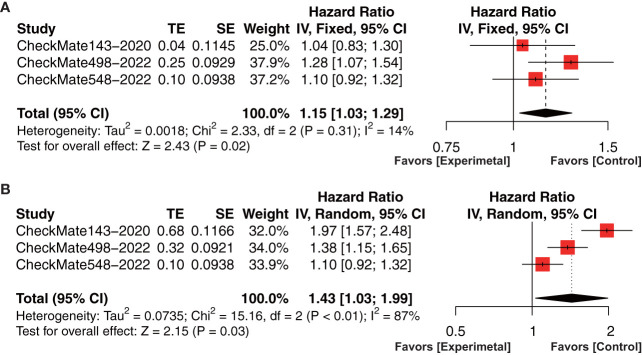
Forest plot showing the effect of anti-PD-1/PD-L1 treatment on **(A)** OS and **(B)** PFS in glioma patients in clinical trials. OS, overall survival; PFS, progression-free survival.

There are 3 phase III RCT studies (CheckMate 143, CheckMate 498, and CheckMate 548), involving 822 patients in the PD-1/PD-L1 inhibitor arm and 823 patients in the control arm, included in this meta-analysis with available data on OS and PFS (26, 32, 33). In the included phase III RCTs, CheckMate 143 compared nivolumab and bevacizumab in recurrent GBM, CheckMate 498 compared nivolumab + RT and TMZ + RT in newly diagnosed GBM, and CheckMate 548 compared nivolumab + RT + TMZ with placebo + RT + TMZ in newly diagnosed GBM (**Table 1**). Forest plot indicated that PD-1/PD-L1 inhibitor could not prolong the OS (HR=1.15, 95% CI: 1.03-1.29, *P*=0.02, I^2 ^= 14%, **Figure 2A**) and PFS (HR=1.43, 95% CI: 1.03-1.99, *P*=0.03, I^2 ^= 87%, **Figure 2B**).

### 3.3 Overall survival by single-arm analysis

**Figure 3:**
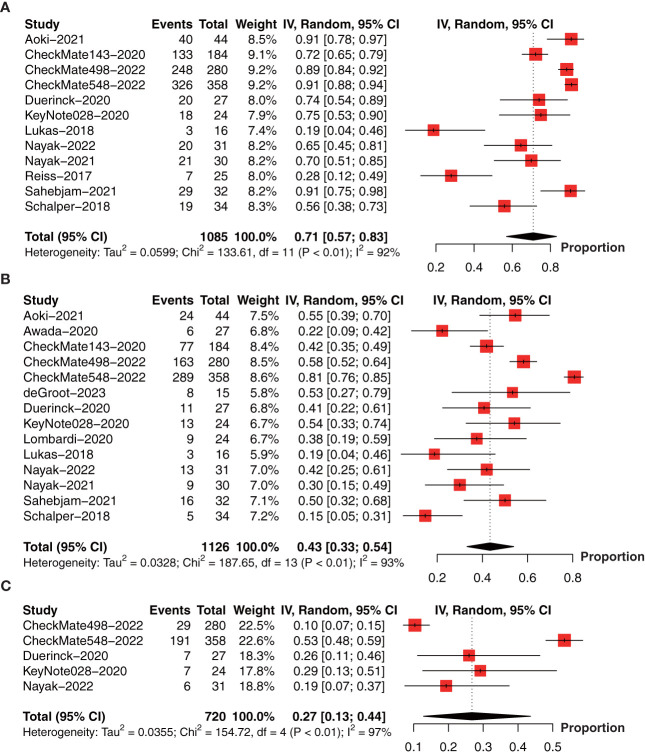
Forest plot showing the **(A)** 6-month, **(B)** 1-year, and **(C)** 2-year overall survival of glioma patients treated with PD-1/PD-L1 inhibitors.

To obtain the OS data for PD-1/PD-L1 inhibitors, we pooled the OS data at 6-month (12 studies with 1085 patients) (18, 23, 26, 28, 30–37), 1-year (14 studies with 1126 patients) (18, 19, 22, 23, 26, 28, 30–37), 2-year (5 studies with 720 patients) (23, 26, 31, 32, 34), and median OS (15 studies with 1552 patients) (18, 19, 22–27, 29–34, 37) in a single arm. The forest plot showed that the 6-month OS rate was 71% (95% CI: 57%-83%, I^2 ^= 92%, *P*<0.01, **Figure 3A**); 1-year OS rate was 43% (95% CI: 33%‐54%, I^2^ = 93%, *P*<0.01, **Figure 3B**), and 2-year OS rate was 27% (95% CI: 13%-44%, I^2 ^= 97%, *P*<0.01, **Figure 3C**).

**Figure 4:**
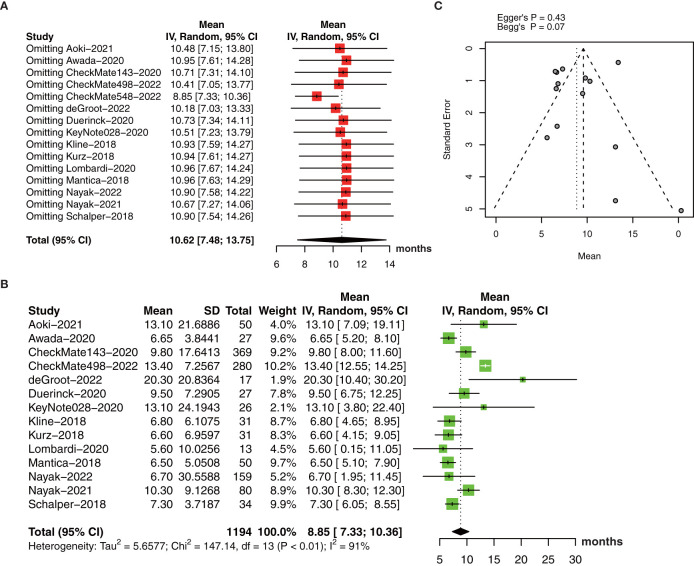
**(A)** The pooled estimate of median OS. **(B)** sensitive analysis. **(C)** the adjusted pooled estimate of median OS. OS, overall survival.

The pooled estimate of median OS was 10.62 months (95% CI: 7.48-13.75, I^2 ^= 95%, *P*<0.01). One study (26) was excluded after the sensitive analysis of median OS (**Figure 4A**). The adjusted pooled estimate of median OS was 8.85 months (95% CI: 7.33-10.36, I^2 ^= 91%, *P*<0.01, **Figure 4B**).

Subgroup analysis revealed study drug, design, and diagnose may be an important source of heterogeneity (**Table 2**). Pembrolizumab showed a longer median OS than nivolumab and other drugs with significant differences between subgroups. In addition, retrospective studies had worse OS than phase I to phase III RCTs with a low heterogeneity. What’s more, anti-PD-1/PD-1 therapy demonstrated a longer OS in primary glioma patients than recurrent glioma patients.

**Table 2:** Subgroup analysis of pooled estimates of the median overall survival.

Subgroup	No. of studies	Mean (months)	95% CI	P value between subgroups	heterogeneity within subgroups		
					I^2^ (%)	P value	
**Drug**				0.04			
Nivolumab	7	9.23	7.10, 11.35		95	< 0.01	
Pembrolizumab	4	11.06	6.04, 16.07		58	0.07	
Others	3	6.64	5.43, 7.85		0	1.0	
**Study Design**				< 0.01			
Phase I	6	9.79	7.15, 12.43		0	0.47	
Phase II	2	8.88	6.60, 11.15		73	< 0.01	
Phase III	2	11.69	8.17, 15.21		92	< 0.01	
Retrospective	4	6.56	5.52,7.59		0	0.98	
**Diagnose**				< 0.01			
Primary	13	13.40	12.55, 14.25		/	/	
Recurrent	1	8.07	6.99, 9.16		62	< 0.01	

The funnel plot for the median OS was symmetric (**Figure 4C**) with no significant differences by Egger’s test (*P*=0.43) and Begg’s test (*P*=0.07).

### 3.4 Progression‐free survival by single-arm analysis

**Figure 5:**
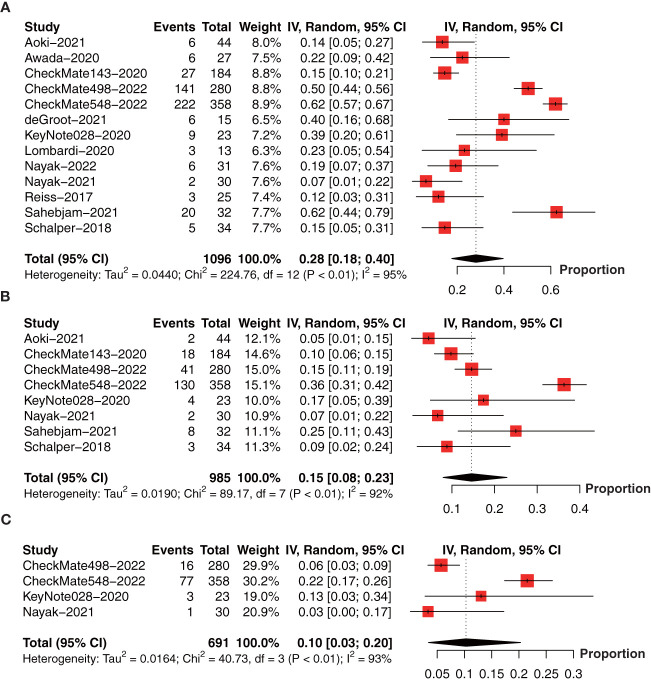
Forest plot showing the **(A)** 6-month, **(B)** 1-year, and **(C)** 18-month progression-free survival in glioma patients receiving anti-PD-1/PD-L1 treatment.

We further collect the PFS data at 6-month (13 studies involving 1096 patients) (18, 19, 22, 26, 27, 30–37), 1-year (8 studies involving 985 patients) (18, 26, 30, 32–34, 36, 37), 18-month (4 studies involving 691 patients) (26, 30, 32, 34), and median PFS (14 studies involving 1523 patients) (18, 19, 22, 24–27, 29–34, 37) in a single arm. The forest plot demonstrated that the 6-month PFS rate was 28% (95% CI: 18%-40%, I^2 ^= 95%, *P*<0.01, **Figure 5A**); 1-year PFS rate was 15% (95% CI: 8%‐23%, I^2^ = 92%, *P*<0.01, **Figure 5B**), and 18-month PFS rate was 10% (95% CI: 3%-20%, I^2 ^= 93%, *P*<0.01, **Figure 5C**).

**Figure 6:**
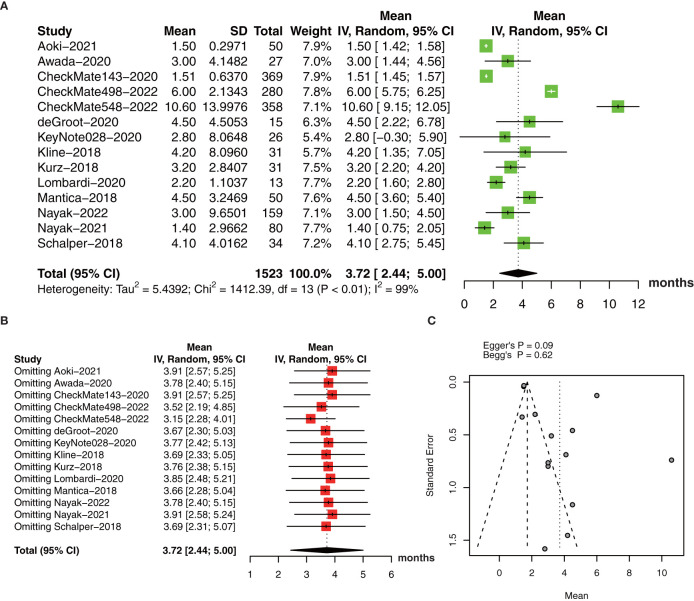
**(A)** The pooled estimate of median PFS. **(B)** sensitive analysis. **(C)** funnel plot. PFS, progression-free survival.

The collective estimate of median PFS was 3.72 months (95% CI: 2.44-5.00, I^2 ^= 99%, *P*<0.01, **Figure 6A**). The sensitivity analysis of median PFS indicated that our outcomes were stable and reliable (**Figure 6B**). Subgroup analysis found the OS of primary glioma patients receiving anti-PD-1/PD-1 therapy was significantly longer than that of recurrent glioma patients, which may be an important source of heterogeneity (**Table 3**). No publication bias was found by funnel plot, Egger’s test (*P*=0.09) and Begg’s test (*P*=0.62) (**Figure 6C**).

**Table 3:** Subgroup analysis of pooled estimates of the median progression-free survival.

Subgroup	No. of studies	Mean (months)	95% CI	P value between subgroups	heterogeneity within subgroups	
					I^2^(%)	P value
**Drug**				0.20		
Nivolumab	7	4.60	2.28, 6.91		100	< 0.01
Pembrolizumab	4	2.33	1.18, 3.48		65	0.04
Others	3	3.11	2.37, 3.84		0	0.97
**Study Design**				0.56		
Phase I	1	2.80	0.30, 5.90		/	/
Phase II	6	2.68	1.63, 3.72		82	< 0.01
Phase III	3	6.00	0.88, 11.12		100	< 0.01
Retrospective	4	3.37	2.20, 4.54		84	< 0.01
**Diagnose**				0.02		
Primary	2	8.24	3.74, 12.75		88	< 0.01
Recurrent	12	2.78	2.06, 3.50		97	< 0.01

### 3.5 Objective response rate by single-arm analysis

**Figure 7:**
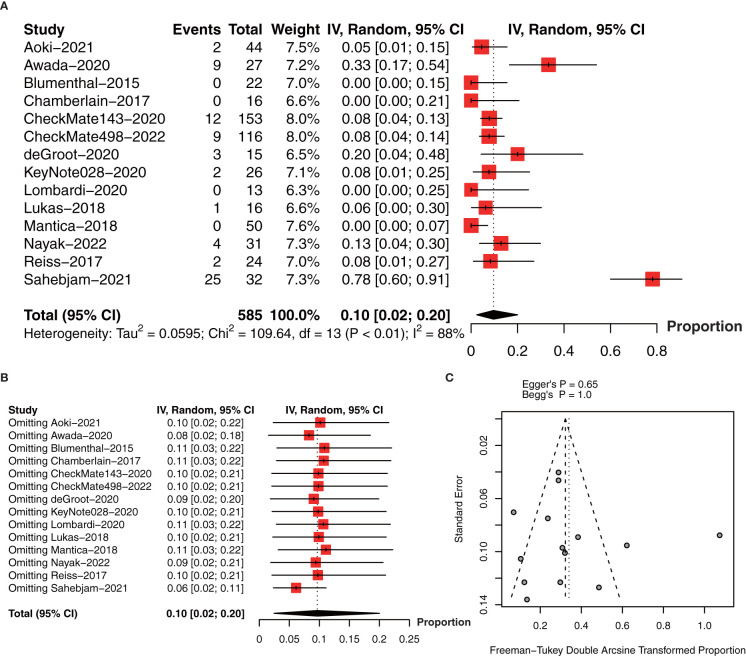
**(A)** The pooled estimate of ORR. **(B)** sensitive analysis of ORR. **(C)** funnel plot of median ORR. ORR, objective response rate.

In terms of ORR, we included 14 studies with 585 patients (18–22, 27–29, 31–36). The pooled estimate of ORR was 10% (95% CI: 2%-20%, I^2^ = 88%, *P*<0.01, **Figure 7A**). The sensitivity analysis and publication bias analysis did not identify the source of heterogeneity (**Figures 7B, C****)**.

### 3.6 Treatment-related adverse events

**Figure 8:**
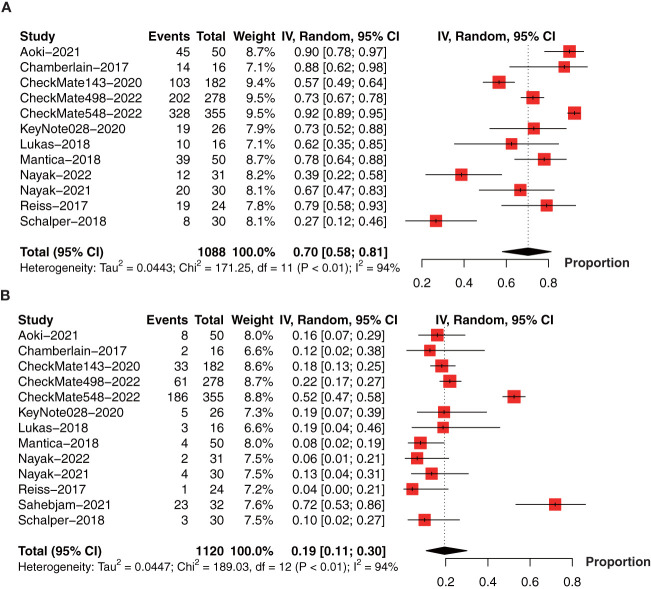
Forest plot showing the incidence of anti-PD-1/PD-L1-related **(A)** AEs and **(B)** AEs ≥ grade 3 in glioma. AEs, adverse events.

To determine the incidence of PD-1/PD-L1 inhibitor-related adverse events (AEs), we performed the meta-analysis of AEs (1088 patients in 12 studies) (18, 21, 26, 28–35, 37) and AEs ≥ grade 3 (1120 patients in 13 studies) (18, 21, 26, 28–37). The pooled incidence of AEs was 70% (95% CI: 58%-81%, I^2 ^= 94%, *P*<0.01, **Figure 8A**). The incidence of AEs ≥ grade 3 was 19% (95% CI: 11%-30%, I^2 ^= 94%, *P*<0.01, **Figure 8B**).

## 4 Discussion

### 4.1 Summary evidence

A total of 20 clinical trials involving 2,321 glioma patients were included in this meta-analysis to evaluate the efficacy and safety of PD-1/PD-1 inhibitors in the treatment of glioma. To our surprise, compared to control arm, anti-PD-1/PD-L1 therapy didn’t prolong OS and PFS but shortened it in Phase III RCTs meta-analysis.

Single-arm analysis indicates that the 6-month OS rate was 71%, 1-year OS rate was 43%, and 2-year OS rate was 27% after anti-PD-1/PD-L1 therapy, and the 6-month PFS rate was 28%, 1-year PFS rate was 15%, and 18-month PFS rate was 10%. The pooled estimate of median OS and PFS in glioma patients received anti-PD-1/PD-L1 therapy was 8.85 months and 3.72 months, respectively. A previously published meta-analysis demonstrated that 6-month OS rate 73%, 1-year OS was 36%, 6-month PFS rate 43%, and 1-year PFS rate was 17% in recurrent GBM patients treated with re-irradiation (38). A randomized Phase III study showed that in newly diagnosed GBM patients receiving TMZ adjuvant RT, 2-year OS rate was 27.2% and median OS was 14.6 months, respectively (39). Moreover, the ORR after anti-PD-1/PD-L1 therapy was only 10%. These evidences suggested that anti-PD-1/PD-L1 could not improve survival in glioma.

Previous single-arm meta-analysis have shown that the incidence of AEs ≥ grade 3 of PD-1/PD-L1 inhibitors was 34.62% in breast cancer (40), 13.4% in head and neck cancer (41), and 21% in lymphoma (42). Our results indicated that in glioma patients treated with PD-1/PD-L1 inhibitors, the incidence of AEs and AEs ≥ grade 3 was 70% and 19%, respectively. In Checkmate 143 phase III RCT, Nivolumab (18.1%) and Bevacizumab (15.2%) had similar rates of AEs ≥ grade 3 (33), and in Checkmate 498 phase III RCT, Nivolumab plus RT (21.9%) *vs* TMZ plus RT (25.1%) exhibited lower AEs ≥ grade 3 (43). It’s worth noting that the combined use of PD-1/PD-L1 inhibitors and chemotherapy had a higher incidence of AEs and AEs ≥ grade 3 in non-small cell lung cancer (44). The results of CheckMate 548 suggested that Nivolumab + RT + TMZ versus Placebo + RT + TMZ treatment significantly increased the incidence of AEs ≥ grade 3 (52.4% *vs*. 33.6%) (45). In newly diagnosed primary GBM, the most common AEs ≥ grade 3 of sequential monotherapy with PD-1/PD-L1 inhibitors include fatigue, pruritus, and immune-mediated AEs (diarrhea, increased alanine aminotransferase and rash), no treatment-related deaths were reported (33). While PD-1/PD-L1 inhibitors combined with RT+TMZ can increase the incidence of AEs ≥ grade 3 and serious AEs leading to discontinuation, which included respiratory failure, respiratory distress, myocarditis, and pneumocystis pneumonia (45). These findings suggest that anti-PD-1/PD-L1 sequential monotherapy is relatively safe in glioma, and serious adverse reactions should be vigilant in combination with other therapies.

### 4.2 Implication

At present, GBM is considered to be an immunosuppressive tumor, which activates various immune escape mechanisms of the tumor microenvironment (TME), including TGF-β and PD-1/PD-L1 (46, 47). Although PD-L1 is overexpressed in glioma, some studies have shown that the expression level of PD-L1 varies greatly among gliomas of different grades and molecular subtypes, ranging from 6.1% to 88% (47). In general, PD-L1 expression was higher in high-grade than in lower-grade gliomas, and PD-L1 expression was most active in mesenchymal gliomas in terms of molecular subtypes (48–50). There is sufficient preclinical evidence to support PD-1/PD-L1 inhibitors as promising potential glioma therapeutics (51). However, the results of previous clinical trials to date have been mixed, with most showing limited therapeutic activity of PD-1/PD-L1 inhibitors in glioma. This may be related to the high tumor heterogeneity and complex TME of glioma patients in the real world, which cannot be accurately simulated by preclinical animal models (52, 53). ORR of anti-PD1/PDL1 therapy was affected by the positive expression of PD1/PD-L1 in tumors. For example, in recurrent cervical cancer, pembrolizumab was observed to have antitumor activity in patients with PD-L1 positive tumors (combined positive score, ≥1), but not in PD-L1 negative tumors (54), and cemiplimab exhibited higher ORR in patients with PD-L1 positive expression than in patients with PD-L1 low expression (55). Notably, in the three included phase III RCTs, the proportion of PD-L1 expression level ≥1% was only 26.1% in CheckMate 143, 37.8% in CheckMate 498, 35.4% in CheckMate 548 in the anti-PD-1 arm, and ORR was 7.8% in CheckMate 143, and 7.8% in CheckMate 498. Therefore, we thought that low PD-L1 expression level may still be an important reason why PD-1/PD-L1 failed to improve the survival in glioma. Mechanically, omics analysis found that glioma patients with effective PD-1/PD-L1 inhibitors treatment were more likely to have mutations in the MAPK pathway, while those with ineffective treatment were enriched with PTEN mutations (56).

Previous studies have suggested that the central nervous system is characterized by blood-brain barrier (BBB), lack of lymphatic drainage system and antigen-presenting cells, and belongs to the “immune privileged zone” (57, 58). More recently, functional lymphatic vessels have been found in the dural sinus between the brain surface and the skull, which are directly connected to the deep cervical lymph nodes (59), suggesting the existence of a structural basis that allows peripheral immune cells to enter the central nervous system. In glioma tissue, the infiltrating CD4^+^ T cells and CD8^+^ T cells were significantly increased (60). These infiltrating T cells in glioma tissue express multiple immune checkpoint molecules and are in a state of exhaustion similar to chronic viral infection, leading to severe T cell dysfunction (61, 62). PD-1/PD-L1 inhibitors can be used in GBM by alleviating T-cell exhaustion through inhibition of immune checkpoint mediated immune escape (22, 53, 62, 63). In this meta-analysis, the ORR after anti-PD-1/PD-L1 therapy was low, which may be not only related to PD-1/PD-L1 expression, but also related to the proportion of immune cell infiltration in gliomas, radiotherapy, and other factors.

The BBB is a physiological barrier that maintains the stability of the physiological environment of the central nervous system and protects the brain tissue from harmful substances. In some clinical trails of brain metastases in patients with melanoma and NSCLC patients, PD-1/PD/L1 inhibitors have shown good efficacy without significant toxicity. This may be due to brain metastases disrupt the BBB and alter its permeability, resulting in increased intracranial drug concentrations (64). At present, some researchers are investigating the use of methods such as intracranial catheters to bypass the BBB for drug delivery (53). It is worth noting that a study of intracranial administration of nivolumab included in our study found that intracranial administration improved the OS of glioma, significantly reduced the dose of drugs used, and reduced the incidence of AEs in patients (23). Therefore, we suggest that future PD-1/PD-L1 clinical treatment trials should consider new drug delivery methods or more appropriate drug combination regimens.

### 4.3 Limitation

Initially, anti-PD-1/PD-L1 therapy is often used in combination with other drugs, but in this meta-analysis, most studies used anti-PD-1/PD-L1 therapy alone. In the CheckMate 498 and CheckMate 548 trials, nivolumab was used in combination with RT and standard RT plus TMZ, but the results were unsatisfactory. However, there are still many therapies worth trying in combination with PD-1/PD-L1, and one of which is intravenous administration before surgery or intracranial administration during surgery combined with standard RT + TMZ. Thus, the efficacy of the combined use of PD-1/PD-L1 inhibitors remains to be explored. Secondly, the PD-1/PD-L1 inhibitors used in the RCTs were nivolumab, and other types of PD-1/PD-L1 inhibitors have not been evaluated in RCTs. Finally, the heterogeneity within our meta-analysis was high due to different study designs, which may have introduced potential bias into our research.

## 5 Conclusion

The results of this meta-analysis suggest that anti-PD-1/PD-L1 therapy is relatively safe but could not prolong survival in glioma. Further investigation are need to confirm this observation.

## Data availability statement

The original contributions presented in the study are included in the article/supplementary material. Further inquiries can be directed to the corresponding author.

## Author contributions

Y-FZ, X-YW and Q-HG drafted the manuscript. Y-FZ and W-JZ designed the study. W-JZ and Z-CG revised the manuscript. X-YW, S-YC, Z-ZL and SD were responsible for the collection of data. YZ was responsible for data analysis. All authors contributed to the article and approved the submitted version.
